# Personal Appearance

**Published:** 1878-01

**Authors:** 


*Personal Appearance and the Culture of Beauty with Hints as to Character.* By T. S. SOZINSKEY, M. D., Ph. D. Philadelphia: Allen, Lane & Scott, 233 South Fifth Street. 1877. pp. 196.

About half a century ago a book on Personal Beauty was written by Dr. John Bell and published in this country. In 1870 another book entitled "Health and the Human Form" was prepared by Drs. Brinton and Napheys of Philadelphia and published by W. J. Holland, Springfield, Mass.

Dr. Sozinskey gives to the public this third and latest of American publications upon this subject written from a physician's stand point. It has been in course of preparation for the last seven years and the author has spared no pains necessary to make it a success. It contains more scientific facts than its latest predecessor, is very prettily gotten up on tinted paper and is in clean type.

But if one wishes to create beauty it can be done more easily and surely as well as more satisfactorily and safely than by the use of pomades or freckle lotions or rouges or depilations. Wholesome habits and industrious and worthy ambitions are the best cosmetics with which we are acquainted and if more is needed a physician's special advice is the proper thing to secure and not the wholesale advice found in newspaper items, quack advertisements and books on "Personal Beauty." M. B. M.

